# Prevalence of depression and its association with burden of care among family caregivers of individuals undergoing cancer treatment at the Kenyatta national hospital cancer treatment centre

**DOI:** 10.1192/j.eurpsy.2026.10656

**Published:** 2026-07-31

**Authors:** S. M. Kimotho, T. MutavI, P. Kigamwa

**Affiliations:** Department Of Psychiatry, Universitty Of Nairobi, Nairobi, Kenya

## Abstract

**Introduction:**

Family caregivers provide critical support to individuals undergoing cancer treatment, often at substantial personal cost. Globally, up to 60% experience psychological distress, yet their needs are rarely addressed. In Kenya, data on caregiver mental health are limited despite the rising cancer burden. This study assessed the prevalence of depression and its association with caregiver burden among caregivers at a national oncology center.

**Objectives:**

To determine depression prevalence and its association with caregiver burden among family caregivers of cancer patients.

**Methods:**

A descriptive cross-sectional study recruited 259 caregivers at Kenyatta National Hospital Cancer Treatment Centre via systematic random sampling over two months. Data were collected using an interviewer-administered questionnaire. Depression was measured using the *Patient Health Questionnaire-9 (PHQ-9)* and caregiver burden with the *Zarit Burden Interview (ZBI).* Descriptive statistics and logistic regression were performed using SPSS v26.

**Results:**

Depression prevalence was 44.0%, mean PHQ-9 score 9.61 (SD ± 6.6). High caregiver burden was reported by 37.8%, mean ZBI score 16.96 (SD ± 12.15). Caregiver burden was the strongest predictor of depression (*p*<0.001); those with no/mild burden were over three times more likely to report no depression (OR=3.317). Depression showed no significant association with age, gender, education, or income.

**Conclusions:**

Depression is common among cancer caregivers in Kenya, driven primarily by caregiver burden rather than socio-demographic factors. Findings highlight the need for routine mental health screening, caregiver-focused psychosocial support, and policy measures to address caregiver strain in oncology care.

**Disclosure of Interest:**

None Declared

